# Phase III randomised chemoprevention study of Selenium on the recurrence of non-invasive bladder cancer. The SELEnium and BLAdder cancer Trial (SELEBLAT)

**DOI:** 10.1186/2049-3258-73-S1-P5

**Published:** 2015-09-17

**Authors:** Maria (Mieke) Goossens, Maurice Zeegers, Hendrik Van Poppel, Steven Joniau, Koen Ackaert, Filip Ameye, Ignace Billiet, Kurt Dillen, Lieven Goeman, Siska Van Bruwaene, Frank Van der Aa, Kris Vekemans, Frank Buntinx

**Affiliations:** 1 KU Leuven, Leuven, Belgium; 2 University Maastricht, Maastricht, Netherlands; 3 UZ Leuven, Leuven, Belgium; 4 Uz Leuven, Leuven, Belgium; 5 Sint-Elisabethziekenhuis, Turnhout, Belgium; 6 AZ Maria Middelares, Gent, Belgium; 7 AZ Groeninge, Kortrijk, Belgium; 8 Jessa ziekenhuis, Hasselt, Belgium; 9 H. Hart ziekenhuis, Roeselaere, Belgium

## Background

In Belgium, bladder cancer is the fifth most common cancer in males (5.2%) and the sixth most frequent cause of death from cancer in males (3.8 %). The per-patient lifetime cost is the highest of all other types of cancer. Previous epidemiological studies have consistently reported that selenium concentrations were inversely associated with the risk of bladder cancer. We therefore hypothesized that selenium may also be suitable for chemoprevention of recurrence.

## Design

The SELEnium and BLAdder cancer Trial (SELEBLAT) was an academic phase III placebo-controlled, double-blind, randomized trial designed to determine the effect of selenium on recurrence of bladder cancer.

## Method

The SELEBLAT study included 277 patients with non-invasive transitional cell carcinoma of the bladder on TURB operation in 16 Belgian hospitals. Patients were randomly assigned to selenium yeast (200 mg/day) supplementation for 3 years or matching placebo, in addition to standard care. All study personnel and participants were blinded to treatment assignment for the duration of the study.

## Results

After an average of 27 months 56 patients, 34 and 22 in treatment group one and two respectively, recurrenced. The Cox regression analysis showed no difference in recurrence-free interval between the two groups (HR = 0.75 (0.44 - 1.28)). Eight patients had progression from a low grade lesion to high grade lesion (HR = 1.89 (95% CI 0.45-7.90)).

## Conclusion

Selenium yeast, in addition to standard care, did not diminish recurrence in bladder cancer patients. Based on the result of the Seleblat study, we do not recommend prescribing selenium to patients in order to prevent recurrence of bladder cancer. Our final results will be pooled with the results of a similar trial in the United Kingdom. This analysis will have sufficient statistical power to be able to confirm or refute our current recommendations.

## Trial registration

ClinicalTrials.gov identifier: NCT00729287

